# Task Sharing Implant Insertion by Community Health Workers: Not Just Can It Work, but How Might It Work Practically and With Impact in the Real World

**DOI:** 10.9745/GHSP-D-15-00230

**Published:** 2015-09-10

**Authors:** Lois Schaefer

**Affiliations:** ^a^​ United States Agency for International Development, Washington, DC, USA

## Abstract

Demonstrating that a health service, such as providing contraceptive implants, can be safely task shared to less highly trained workers is crucial but is only one step toward effective implementation at scale. Providers need dedicated time, enough clients, supplies, supervision, and other system support, allowing them to maintain their competency, confidence, and productivity.

See related article by Charyeva.

Task shifting is defined as “the rational redistribution of tasks among health workforce teams. Specific tasks are moved, where appropriate, from more highly qualified health workers to health workers with shorter training and fewer qualifications, in order to make more efficient use of the available human resources for health.”^1^ It is becoming more commonly known as task sharing, a change intended to convey the message that tasks are not taken away from one cadre and given to another, but rather that additional cadres are given the capacity to take on identified tasks.

Most commonly, task sharing has been used to shift skills from nurses, midwives, and doctors to community health workers (CHWs), in order to increase the availability of selected services, including family planning, child health monitoring, and postpartum follow-up, at the community level. However, task sharing is increasingly being implemented with mid-level providers to increase access to clinical skills, such as insertion of contraceptive implants or provision of antiretroviral therapy, by sharing such tasks from physicians to nurses and midwives. It has also been used to create new cadres of workers, for example, clinical officers who can provide cesarean deliveries or other simple surgeries, when there is no appropriate existing cadre.

## EFFECTIVE IMPLEMENTATION AT SCALE?

Most frequently, the question that is addressed in pilots or other studies is whether a cadre can safely and competently complete the task that is to be shared—for example, can CHWs learn to give injectable contraceptives? Or, as in the case of an operations research project described by Charyeva and colleagues in this issue of GHSP,^2^ can community health extension workers (CHEWs) in northern Nigeria insert and remove implants? The design of such task sharing interventions focuses on demonstrating that there is no harmful effect on recipients of those services. But documenting that a worker can safely provide a service is only one of the questions that need to be answered for safe, efficient, effective, equitable, and sustainable implementation of task sharing.

### Context Matters

Of crucial importance is the context or environment in which task sharing takes place. First and foremost, health workers must receive adequate preparation for assuming the new tasks, which often includes competency-based training, and then be supported by supervision, mentoring, and functional referral systems as they provide services. In general, they must also receive adequate incentives and compensation for the new workload, something that is often overlooked and underestimated for building worker motivation and performance. They must be offered adequate legal protection for their new roles through updated laws, regulations, policies, and guidelines. Finally, they must operate in a service delivery system organized to be conducive to large-scale effect.

Few countries provide such an environment, however, and so efforts to introduce and pilot task sharing often address creating an enabling environment. But concerns for safety and proving feasibility often mean that the intervention is designed in such a way that, while it leads to a successful pilot, it is not replicable in the resource-limited reality of most countries. Intensive, specialized training courses are beyond the capacity of local training systems, for example, or post-training supervision is of a frequency and intensity that is beyond what the standard supervision system can provide. Worker incentives that the health system cannot absorb or sustain are often used to improve motivation in pilot settings. Although this may be necessary to achieve that first important step of demonstrating that a cadre can, in fact, take on a new skill or task, it does not provide a model that can be replicated, much less scaled-up.

Task sharing pilots tend to be designed in such a way that they are not replicable in resource-limited settings.

### Making Task Sharing More Practicable

Moving forward, task sharing interventions need to be designed more carefully, with an eye to local capacity for ongoing implementation. This begins with a strong understanding and acknowledgment of the specific service delivery context and includes plans for transfer of activities to local partners, whenever possible. In the design stage, selecting interventions that are feasible and sustainable should be a primary consideration. Providing a clear and detailed description of the pre-project context when documenting the experience will help others assess the feasibility of the project’s interventions in other settings. Discussion of the results needs to demonstrate thinking that goes beyond project mode and includes a hard look at how the intervention could go to scale and be sustained.

## RELEVANCE FOR PROVISION OF IMPLANTS

In this issue of GHSP, Charyeva and colleagues clearly present the need to increase the number of health workers able to provide contraceptive implants, as part of a larger reasonable emphasis on increasing access to long-acting and permanent methods.^2^ The authors provide a description of how such services are currently provided and effectively convey the role for CHEWs in achieving that goal. For example, they make sure the reader knows what CHEWs are—how they are trained, their current scope of work, and how they fit into the health system—important information for replication, given that the definition of CHWs varies widely and that these factors affect their ability to assume new skills.

The authors are less successful, however, in addressing the feasibility and sustainability of their project interventions. Their model for task sharing was based on 2 to 3 weeks of training for the CHEWs, followed by high-frequency supervision—from 1 to 8 visits within the following 6 months—a level that the project itself struggled to provide and which the existing supervision system cannot maintain. The CHEWs interviewed post-intervention acknowledged the usefulness of the supervision—and requested it be increased to 1 to 2 times per month, a level even farther removed from local reality. Although the authors identify the need for “continuous supportive supervision” as a lesson learned, they unfortunately do not offer any insights on how that could be achieved.

Most importantly, the sum total output of this intensive intervention was only 4 implants per health facility per month, providing little confidence of a pathway to large-scale effect. In contrast, other approaches to implant provision such as mobile outreach and social franchising are providing more implants by many orders of magnitude.^3^^-^^6^ Clearly, issues such as the need for demand creation may have limited provision, but organization of work and system demands may be another major constraint. To successfully integrate a service, especially one requiring specialized skills such as implants, requires that providers have dedicated time, all the necessary supplies, and sufficient opportunities to use their skills so they can maintain their competency and confidence. While the CHEWs in the intervention were able to perform 4 insertions per month without adding significantly to their workload, and special systems kept them stocked with implants, this may not be true post-project and with the desired increased demand. This issue could prove an insurmountable constraint on this approach to providing implants.

Successful task sharing of implants requires providers with dedicated time, supplies, and enough client demand to maintain competency.

The authors’ finesse of issues related to scale-up and sustainability with the statement, “Future investigation should rigorously examine factors that support scale-up and sustainability of the intervention,” leaves many unanswered questions. I applaud the work of Charyeva and colleagues as a sound initial “proof of concept,” but we must have more robust discussion on how to fully implement task sharing across contexts from all who share similar results.
